# Novel *HADHB* mutations in a patient with mitochondrial trifunctional protein deficiency

**DOI:** 10.1038/s41439-020-0097-z

**Published:** 2020-04-02

**Authors:** Mina Nakama, Hideo Sasai, Mitsuru Kubota, Yuki Hasegawa, Ryoji Fujiki, Torayuki Okuyama, Osamu Ohara, Toshiyuki Fukao

**Affiliations:** 1 Clinical Genetics Center, Gifu University Hospital, Gifu, Japan; 2 Department of Pediatrics, Graduate School of Medicine, Gifu University, Gifu, Japan; 3 Department of General Pediatrics & Interdisciplinary Medicine, National Center for Child Health and Development, Tokyo, Japan; 4 Department of Pediatrics, Faculty of Medicine, Shimane University, Shimane, Japan; 5 Department of Applied Genomics, Kazusa DNA Research Institute, Chiba, Japan; 6 Department of Clinical Laboratory Medicine, National Center for Child Health and Development, Tokyo, Japan

**Keywords:** Genetics research, Metabolic disorders

## Abstract

We encountered a patient with mitochondrial trifunctional protein deficiency in whom the corresponding mutations were not identified by a DNA panel for newborn screening for targeted diseases. After diagnosis confirmation by an enzyme assay and immunoblotting using the autopsied liver, the re-evaluation of the panel data indicated a heterozygous deletion of exons 6–9 that was later confirmed at the genomic level. cDNA analysis also identified exonization of the 5′ region of intron 9 caused by a deep intronic mutation, c.811 + 82A>G.

## Introduction

Mitochondrial trifunctional protein (TFP) deficiency is a rare, autosomal recessive disorder. The main manifestations of TFP are cardiomyopathy, hypoketotic hypoglycemia, metabolic acidosis, sudden infant death, metabolic encephalopathy, liver dysfunction, peripheral neuropathy, exercise-induced myoglobinuria, and rhabdomyolysis^1,2^. This disease is classified into three subtypes based on the severity and onset age: the most severe form is the lethal phenotype with neonatal onset, the intermediate form is the hepatic phenotype with infant onset, and the mild form is the myopathic phenotype with late-adolescent onset^3^. TFP is a multienzyme complex consisting of four α and four β subunits encoded by the genes *HADHA* (MIM 600890) and *HADHB* (MIM 143450), respectively^4,5^. This complex exhibits three distinct enzyme activities, functioning as a long-chain enoyl-CoA hydratase (LCEH), a long-chain 3-hydroxyacyl-CoA dehydrogenase (LCHAD), and a long-chain 3-ketoacyl-CoA thiolase (LCKT). The α subunit harbors the LCHAD and LCEH functions, and the β subunit exhibits the LCKT function. TFP binds to the inner mitochondrial membrane and plays a significant role in the last three steps of the β-oxidation cycle of long-chain acyl-CoAs^5,6^. The incidence of TFP deficiency has been estimated to be 1 per 100,000 births in Europe^7^. To date, 14 TFP-deficient patients have been reported in Japan^8^. Seventy-two mutations have been identified in *HADHA*, and sixty-seven mutations have been found in *HADHB* (HGMD-professional-release-2019.3). We referred to the HGMD-Professional-release-2019.3 database and ClinVar_20191202 to determine whether the mutations identified in this patient were novel.

The patient was a first child born after 37 weeks and 6 days of gestation by cesarean section to nonconsanguineous parents. His birth weight was 2740 g. Newborn screening showed elevated C16-OH acylcarnitine (1.55 nmol/ml; cutoff < 0.1). Subsequent serum acylcarnitine analysis showed the C16-OH level to be 0.221 nmol/ml, with C18:1-OH at 0.219 nmol/ml. Urinary organic acid analysis was not specific. The patient was tentatively diagnosed with TFP deficiency (the patient’s clinical details are described in Supplementary Information 1). Informed consent for molecular analysis obtained from the parents, and the Ethics Committee of Gifu University Hospital approved the study (approval 29–210). Gene panel analysis using next-generation sequencing (NGS) with the MiSeq Sequencing System (Illumina, San Diego, CA, USA) was performed at the Kazusa DNA Research Institute. Genomic DNA was extracted from the patient’s peripheral blood leukocytes. We designed panel consisting of 60 genes was to detect *HADHB* mutations and metabolic disorders (Supplementary Table 1). This panel was designed to capture the designated protein-coding regions and 10 bp of flanking intronic sequences^9^. However, the gene panel analysis did not initially identify any mutations in *HADHA* and *HADHB* or in other genes related to fatty acid beta-oxidation. The patient was carefully managed but died of cardiomyopathy at 3 years 9 months of age after recurrent episodes of rhabdomyolysis.

We confirmed the diagnosis of TFP deficiency based on the lack of TFP enzyme activity and the absence of both HADHA and HADHB proteins in the liver at autopsy (Supplementary Fig. 1). Western blotting was performed using rabbit polyclonal antibodies raised against purified MTP protein as the primary antibody (provided by Dr. T. Hashimoto), as previously described^10^. 3-ketoacyl-CoA thiolase activity, measured as described previously, was almost null in the liver of the patient (1.85 µmol/min/mg protein, control sample 93.9 µmol/min/mg protein)^11^.

**Fig. 1:**
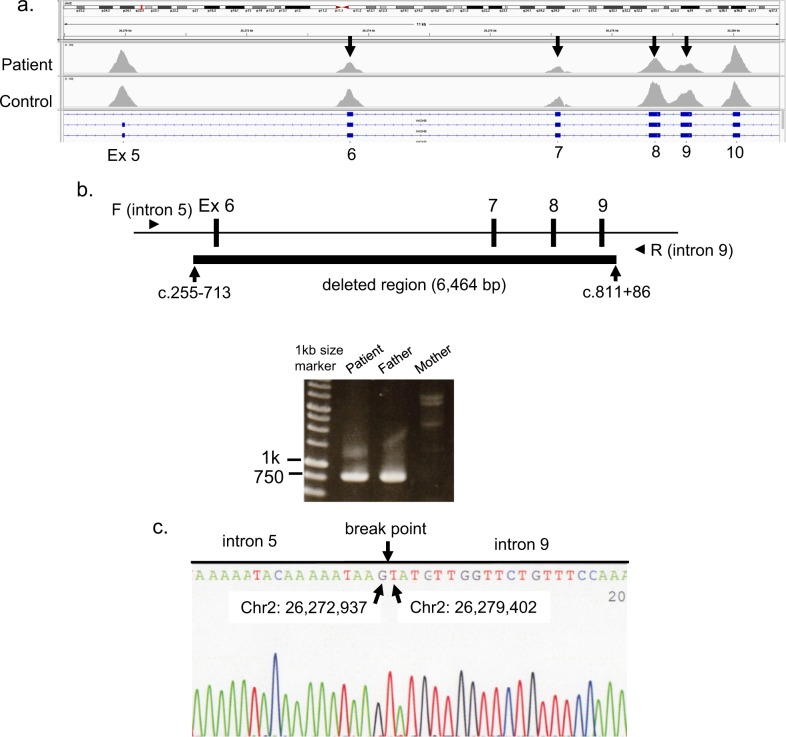
Identified *HADHB* exons 6–9 genomic deletion. **a** Representation of the next-generation sequencing data coverage depth using the Integrative Genomics Viewer. A suspected heterozygous deletion of *HADHB* exons 6–9 was detected. **b** Deletion allele-specific PCR of the *HADHB* gene was performed. The deletion allele could only be amplified by the indicated primers flanking the deletion in the patient and his father. The wild-type allele was too large to be amplified by these primers under the applied PCR conditions; hence, no amplification products were obtained for the patient’s mother. **c** Sequencing analysis of the 781-bp fragment spanning the exons 6–9 deletion indicated breakpoints upstream of exon 6 (c.255–713) and downstream of exon 9 (c.811 + 86) in the *HADHB* gene in both the patient and his father.

Therefore, we re-examined the gene panel data and found that the normalized read depths for exons 6–9 were lower than those of the other exons in *HADHB* (Fig. 1a). Polymerase chain reaction (PCR) using a forward primer for intron 5 (5′-TTCTGGACCTGGTATCAGTC-3′) and a reverse primer for intron 9 (5′-CTCTATGGAACCACAAGCCTT-3′) successfully amplified a truncated genomic fragment (781 bp) in the patient and his father (Fig. 1b). Therefore, the deletion allele of the patient was inherited from his father. Direct sequencing of this fragment revealed the breakpoint (Chr2: 26,272,937–26,279,402 in GRCh38.p13) for the deletion of exons 6–9 (Fig. 1c).

**Fig. 2:**
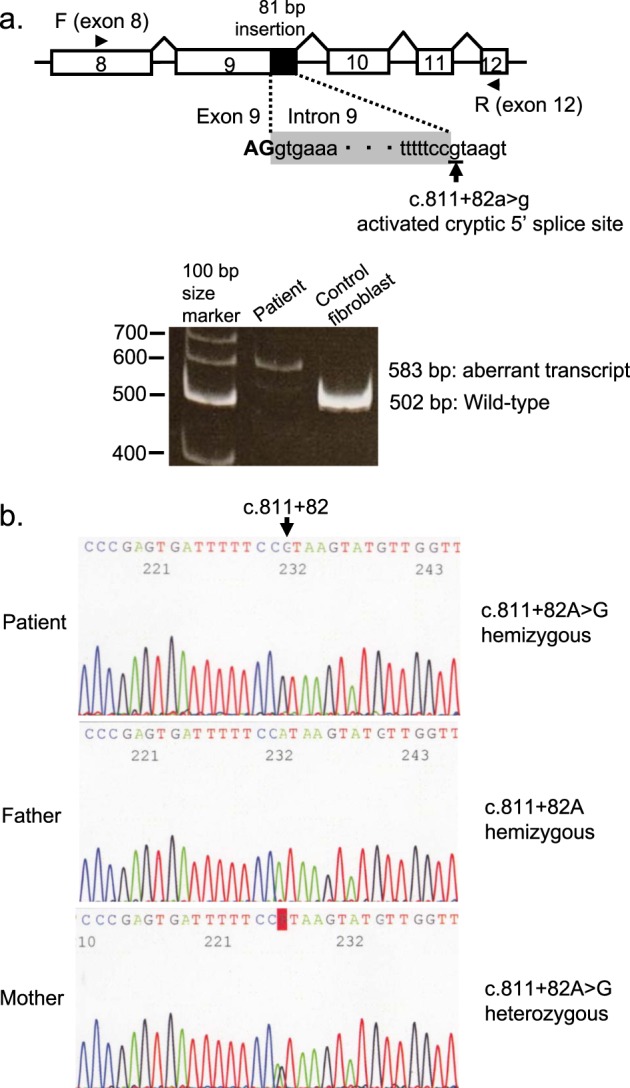
Intronic mutation-induced splicing abnormality in the patient. **a** Schematic representation of the strategy used to detect aberrant *HADHB* transcripts by PCR using a forward primer for exon 8 and a reverse primer for exon 12. The intronic exonized region is shown in the black box (insertion of 81 bp in intron 9). The c.811 + 82A>G variant creates a new cryptic 5′ splice site. Amplified transcripts from the patient were characterized by predominance of the 81-bp sequence of *HADHB*, resulting in a 583-bp amplicon. Transcripts from the control fibroblasts showed only the predicted wild-type amplicon size of 502 bp. **b** The patient and his mother carried the c.811 + 82A>G *HADHB* mutation. The other allele of the patient contained the exons 6–9 deletion, whereas the mother was heterozygous for c.811 + 82A>G and did not carry the deletion. The father was hemizygous for wild-type c.811 + 82A because his other allele contained the exons 6–9 deletion.

To identify the patient’s other mutation inherited from his mother, cDNA analysis was performed. RNA was isolated from the patient’s autopsied heart and a control fibroblast sample (Kurabo, Osaka, Japan) with an Isogen kit (Nippon Gene, Tokyo, Japan). cDNA synthesis was performed using the *HADHB*-specific antisense primers Ex16rv1 (5′-GGATAAGCTTCCACTATCATAGC-3′) and Ex16rv2 (5′-GGCAAGGCTTAAGTGCAAAC-3′), an oligo (dT) primer (Thermo Fisher Scientific, Waltham, MA, USA), and a random hexamer primer (Thermo Fisher Scientific). When cDNA amplification was carried out with a forward primer for exon 8 (5′-ATGCTTGATCTCAATAAGGCC-3′) and a reverse primer for exon 12 (5′-GATCTTTTGGATCCTGAGACAC-3′), a longer cDNA fragment than that of the control was detected (Fig. 2a). Direct sequencing of the larger PCR fragment from the patient showed an insertion (81 bp) between exons 9 and 10. The insertion was derived from intron 9 and introduced a frameshift and premature termination within the insertion (p.Pro270Profs*14).

To shed light on the cause of the exonization of this intronic sequence, we performed direct sequencing of intron 9 and detected a *HADHB* NM_000183.3:c.811 + 82A>G substitution at the genomic DNA level (Fig. 2b). His mother also carried this *HADHB* c.811 + 82A>G mutation in a heterozygous manner. The exonization can be explained by the activation of a cryptic splice site 81 bp downstream of the authentic 5′ splice site of intron 9. This intronic mutation was found in dbSNP (https://www.ncbi.nlm.nih.gov/snp/) in rs757303269 and identified as an intron_variant (minor allele frequency from TOPMED is G = 0.000016/2).

In silico analyses were then performed on the splice sites. MaxEntScan::score5ss (http://hollywood.mit.edu/burgelab/maxent/Xmaxentscan_scoreseq.html) provided a much higher score for the activated cryptic 5′ splice site than for the authentic 5′ splice site of intron 9. The Senapathy & Shapiro (S&S) matrix^12^ and Human Splicing Finder (http://www.umd.be/HSF/HSF.shtml) provided similar scores for the two sites (Supplementary Table 2).

Even though the aberrantly spliced mRNA with a premature termination within the 81-bp insertion should theoretically be subjected to nonsense-mediated mRNA decay, cDNA analysis showed that the aberrantly spliced mRNA was a major transcript in the patient (Fig. 2a). Indeed, the in silico data indicated the predominant use of the activated cryptic 5′ splice site within the patient’s transcripts. Another deep intronic mutation (g.33627A>G) in *HADHB* resulting in intronic exonization was previously found in a Japanese patient^13^.

In conclusion, we report a case of TFP deficiency in a 3-year-old Japanese boy with a new pathogenic *HADHB* intronic mutation resulting in an atypical splice site and a large deletion. Thus, cDNA analysis can provide clues for revealing deep intronic mutations that are difficult to identify by exome or gene panel analysis.

## Supplementary information

Supplementary Figure 1 Western blots of mitochondrial trifunctional protein (TFP) in the liver

Supplementary Table 1 The 60 target genes of our DNA panel

Supplementary Information 1 Clinical details of the patient

Supplementary Table 2 Splice site prediction using in silico tools

## Data Availability

The relevant data from this Data Report are hosted at the Human Genome Variation Database at 10.6084/m9.figshare.hgv.2817. 10.6084/m9.figshare.hgv.2820.

## Data availability

The data and materials described in this report are available upon request. Supplementary Information is available online through the Human Genome Variation journal.
