# Supplementary material for: Novel HADHB mutations in a patient with mitochondrial trifunctional protein deficiency
Source: Hum Genome Var. 2020 Apr 2;7:10. doi: 10.1038/s41439-020-0097-z (PMC7118068; doi:10.1038/s41439-020-0097-z)
Supplement: Supplementary file 1 — Supplementary Figure 1 Western blots of mitochondrial trifunctional protein (TFP) in the liver [file 41439_2020_97_MOESM1_ESM.pptx]

## Slide 1
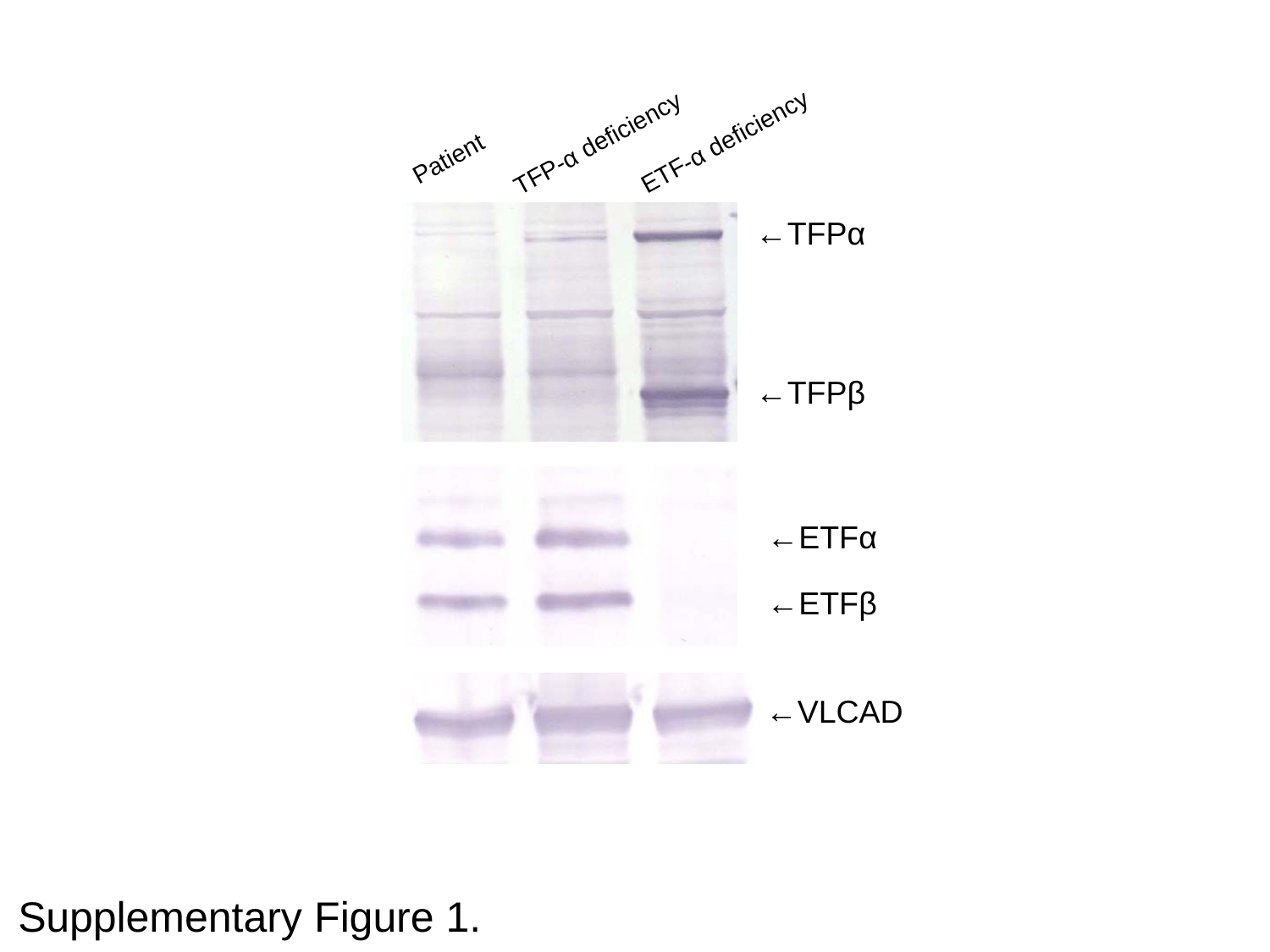

ETF-α deficiency
TFP-α deficiency
Patient
←TFPα
←TFPβ
←ETFα
←ETFβ
←VLCAD
Supplementary Figure 1.
