# Supplementary material for: Novel HADHB mutations in a patient with mitochondrial trifunctional protein deficiency
Source: Hum Genome Var. 2020 Apr 2;7:10. doi: 10.1038/s41439-020-0097-z (PMC7118068; doi:10.1038/s41439-020-0097-z)
Supplement: Supplementary file 2 — Supplementary Table 1 The 60 target genes of our DNA panel [file 41439_2020_97_MOESM2_ESM.docx]

**Supplementary Table 1 Target 60 genes of our panel**

| *GALE* | *CBS* | *LMBRD1* | *ASS1* | *PAH* | *SLC52A2* |
| --- | --- | --- | --- | --- | --- |
| *GALK1* | *MAT1A* | *MMAA* | *BCKDHA* | *HMGCL* | *SLC52A3* |
| *GALT* | *MTHFR* | *MMAB* | *BCKDHB* | *HSD17B10* | *ETFA* |
| *SLC25A20* | *MTR* | *MMACHC* | *DBT* | *ACADM* | *ETFB* |
| *CPT1A* | *MTRR* | *MMADHC* | *DLD* | *SLC22A5* | *ETFDH* |
| *CPT2* | *BTD* | *MUT* | *GCH1* | *HADHA* | *TAZ* |
| *IVD* | *HLCS* | *NAGS* | *PCBD1* | *HADHB* | *AUH* |
| *SLC25A13* | *SLC5A6* | *OTC* | *PTS* | *ACADVL* | *MCCC1* |
| *PCCA* | *ABCD4* | *ASL* | *QDPR* | *GCDH* | *MCCC2* |
| *PCCB* | *HCFC1* | *CPS1* | *SPR* | *SLC52A1* | *ACAT1* |

described as a gene symbol
