# Supplementary material for: Novel HADHB mutations in a patient with mitochondrial trifunctional protein deficiency
Source: Hum Genome Var. 2020 Apr 2;7:10. doi: 10.1038/s41439-020-0097-z (PMC7118068; doi:10.1038/s41439-020-0097-z)
Supplement: Supplementary file 3 — Supplementary Information 1 Clinical details of the patient [file 41439_2020_97_MOESM3_ESM.docx]

**Supplementary Information 1 Clinical details of the patient**

The patient was tentatively diagnosed as having mitochondrial trifunctional protein (TFP) deficiency. Treatment with medium-chain triglyceride formula and bezafibrate was initiated. Nevertheless, laboratory data indicated high serum transaminase (AST 70-140 IU/L) and creatine kinase (CK; 300-1000 IU/L) levels, with no metabolic abnormalities such as hypoketotic hypoglycemia or severe liver dysfunction. At 7 months of age, his CK level markedly increased to 30,367 U/L while he was infected with human metapneumovirus. Rhabdomyolysis is often observed in patients with high CK levels (> 30,000 IU/L) during hospitalization for norovirus or respiratory syncytial virus infections. However, hypoglycemia was not observed in this patient. His CK baseline had risen to 2,000–8,000 U/L from 2 years of age, but he had no symptoms such as muscle weakness. His motor skills began weakening at 3 years of age. He suffered from cardiomyopathy, and despite intensive care using extra-corporeal membrane oxygenation, he died suddenly at 3 years 9 months of age.
