# Supplementary material for: Novel HADHB mutations in a patient with mitochondrial trifunctional protein deficiency
Source: Hum Genome Var. 2020 Apr 2;7:10. doi: 10.1038/s41439-020-0097-z (PMC7118068; doi:10.1038/s41439-020-0097-z)
Supplement: Supplementary file 4 — Supplementary Table 2 Splice site prediction using in silico tools [file 41439_2020_97_MOESM4_ESM.docx]

**Supplementary Table 2 Splice site prediction *in silico* tools**

|  | MaxEntScan (0–16) | HSF (0–100) | S&S (0–84.3) |
| --- | --- | --- | --- |
| c.811  (authentic 5’ss) | 5.07 | 84.34 | 76.64 |
| c.811+82A  (cryptic 5’ss) | 2.09 | 55.42 | 60.04 |
| c.811+82A>G  (activated cryptic 5’ss) | 10.27 | 82.26 | 78.28 |

HSF: Human Splicing Finder, S&S: Senapathy & Shapiro matrix
